# Modulation of circadian clock by crude drug extracts used in Japanese Kampo medicine

**DOI:** 10.1038/s41598-021-00499-w

**Published:** 2021-10-26

**Authors:** Manhui Zhang, Kohei Kobayashi, Haruki Atsumi, Yuma Katada, Yusuke Nakane, Junfeng Chen, Ryo Nagano, Naoya Kadofusa, Taeko Nishiwaki-Ohkawa, Naohiro Kon, Tsuyoshi Hirota, Ayato Sato, Toshiaki Makino, Takashi Yoshimura

**Affiliations:** 1 Institute of Transformative Bio-Molecules (WPI-ITbM), Nagoya University, Furo-cho, Chikusa-ku, Nagoya, 464-8601 Japan; 2 Laboratory of Animal Integrative Physiology, Graduate School of Bioagricultural Sciences, Nagoya University, Furo-cho, Chikusa-ku, Nagoya, 464-8601 Japan; 3 Department of Pharmacognosy, Graduate School of Pharmaceutical Sciences, Nagoya City University, 3-1 Tanabe-Dori, Mizuho-ku, Nagoya, 467-8603 Japan

**Keywords:** Circadian regulation, Circadian mechanisms

## Abstract

Circadian rhythm is an approximately 24 h endogenous biological rhythm. Chronic disruption of the circadian clock leads to an increased risk of diabetes, cardiovascular disease, and cancer. Hence, it is important to develop circadian clock modulators. Natural organisms are a good source of several medicines currently in use. Crude drugs used in Japanese traditional Kampo medicine or folk medicines are an excellent source for drug discovery. Furthermore, identifying new functions for existing drugs, known as the drug repositioning approach, is a popular and powerful tool. In this study, we screened 137 crude drug extracts to act as circadian clock modulators in human U2OS cells stably expressing the clock reporter *Bmal1-dLuc*, and approximately 12% of these modulated the circadian rhythm. We further examined the effects of several crude drugs in Rat-1 fibroblasts stably expressing *Per2-luc*, explant culture of lung from *Per2::Luciferase* knockin mice, and zebrafish larvae in vivo. Notably, more than half of the major ingredients of these crude drugs were reported to target AKT and its relevant signaling pathways. As expected, analysis of the major ingredients targeting AKT signaling confirmed the circadian clock-modulating effects. Furthermore, activator and inhibitor of AKT, and triple knockdown of *AKT* isoforms by siRNA also modulated the circadian rhythm. This study, by employing the drug repositioning approach, shows that Kampo medicines are a useful source for the identification of underlying mechanisms of circadian clock modulators and could potentially be used in the treatment of circadian clock disruption.

## Introduction

The circadian rhythm is an approximately 24 h cell-autonomous biological rhythm observed in virtually all living organisms. The mammalian circadian clock involves a transcription-translation feedback loop in which CLOCK and BMAL1 heterodimers activate the *PERIOD* (*PER*) and *CRYPTOCHROME* (*CRY*) genes, which then provide feedback and repress their transcriptions^1,2^. It has been reported that chronic circadian disruption is associated with many diseases, such as cardiovascular and cerebrovascular diseases^3^, cancer^4^, immune^5^, and metabolic disorders^6^.

The development of new pharmaceuticals is time-consuming and expensive. Therefore, identifying new functions for existing drugs, known as the drug repositioning approach, is a popular and powerful approach^7^. Crude drugs used in traditional Japanese Kampo medicine are the major sources of new chemical entities for drug discovery^8^. In this study, we screened 137 crude drug extracts to identify circadian clock modulators using a human osteosarcoma U2OS cell line stably expressing the clock reporter *Bmal1-dLuc* and identified 17 hit crude drugs. We noticed that more than half of the major ingredients of these crude drugs targeted AKT and relevant signaling pathways. Therefore, we examined the effects of the major ingredients targeted AKT. We then examined the effects of AKT activator and inhibitor, as well as the triple knockdown of AKT1/2/3 using siRNA.

## Results

### Identification of crude drug extracts that modulate the circadian clock

**Figure 1:**
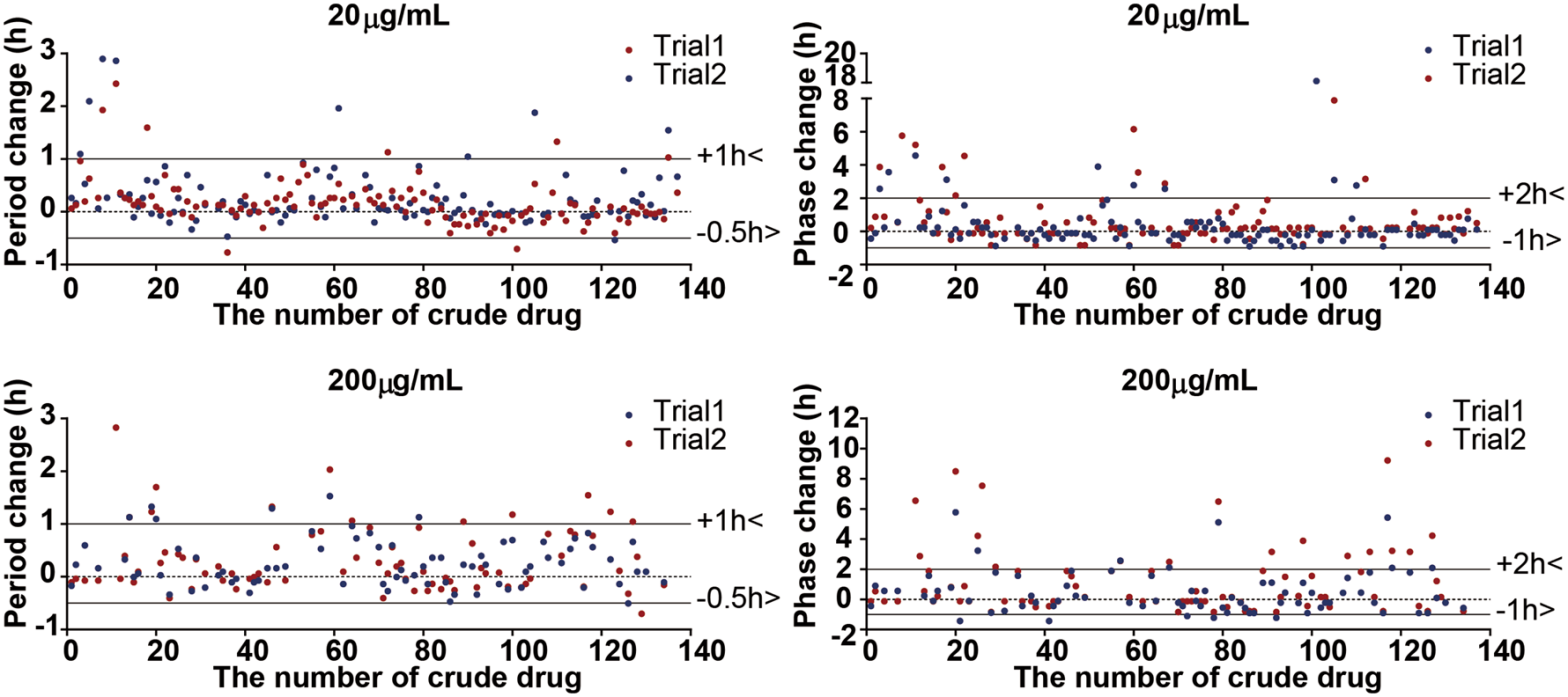
Screening of crude drug extracts for circadian clock modulators. A total of 137 crude drug extracts were screened in *Bmal1-dluc* U2OS cells at 20 µg/mL (top) and 0.20 mg/mL (bottom) for circadian period (left) and phase (right) changes. (Left) Two independent trials identified two crude drugs at 20 µg/mL and four crude drugs at 0.20 mg/mL that show consistent period lengthening effects by 1 or more hours. (Right) This screening also identified five crude drugs at 20 µg/mL and eight crude drugs at 0.20 mg/mL that show consistent phase changing effects by 2 or more hours.

**Figure 2:**
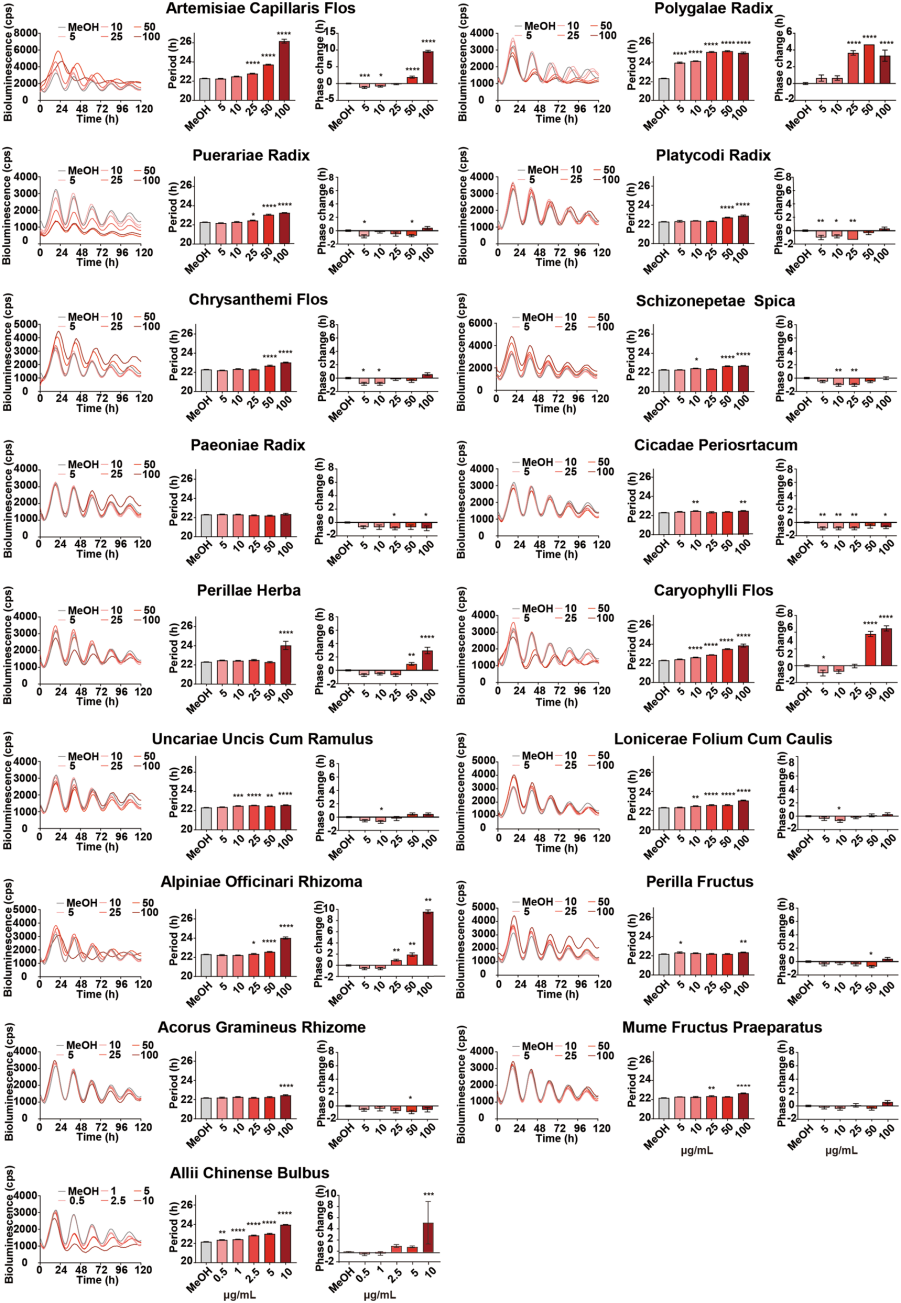
Dose-dependent effect of 17 hit crude drug extracts in U2OS cells expressing *Bmal1-dLuc*. Luminescent traces (left) and dose-dependent effects on the circadian period (middle) and phase (right) in U2OS cells. Values are averages of six replicates ± SD and were analyzed using one way ANOVA, followed by Dunnett’s multiple comparisons test (*p < 0.05, **p < 0.01, ***p < 0.001, **** *p* < 0.0001).

**Figure 3:**
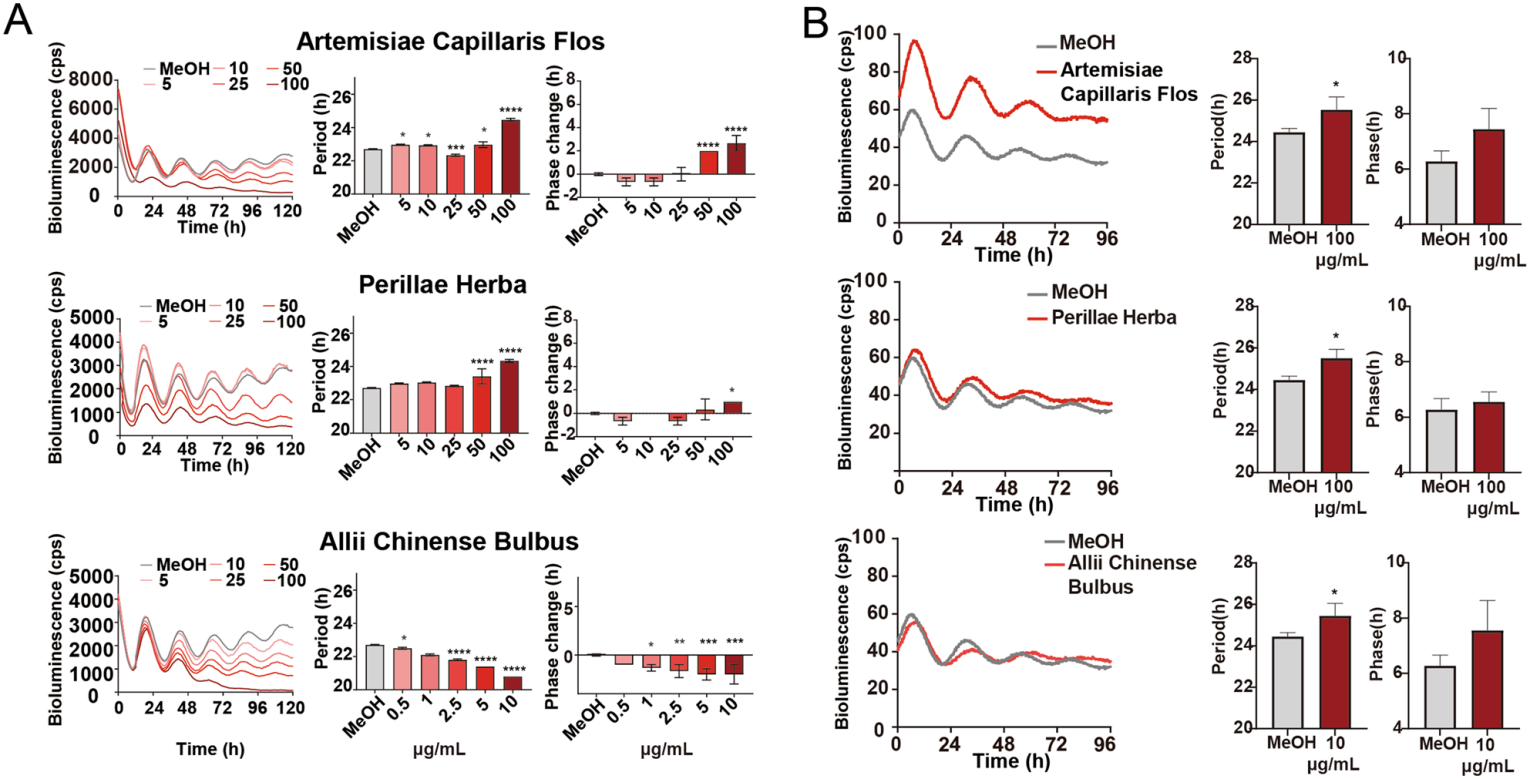
Effect of three crude drug extracts on Rat-1 fibroblasts expressing *Per2-luc* and lung explants from *Per2::Luciferase* knockin mice. (**A**) Luminescent traces (left) and dose-dependent effects on the circadian period (middle) and phase (right) in Rat-1 cells. Values are averages of three replicates ± SD and were analyzed using one-way ANOVA, followed by Dunnett’s multiple comparisons test (*p < 0.05, **p < 0.01, ***p < 0.001, **** *p* < 0.0001). (**B**) Effects of the extracts of Artemisiae Capillaris Flos (top), Perillae Herba (middle), and Allii Chinense Bulbus (bottom) on lung explants of *Per2::Luciferase* knockin mice. Luminescent traces (left) and effects on period (middle) and phase (right). Values are averages of three replicates ± SD and were analyzed using Student's t-test. (*p < 0.05).

**Figure 4:**
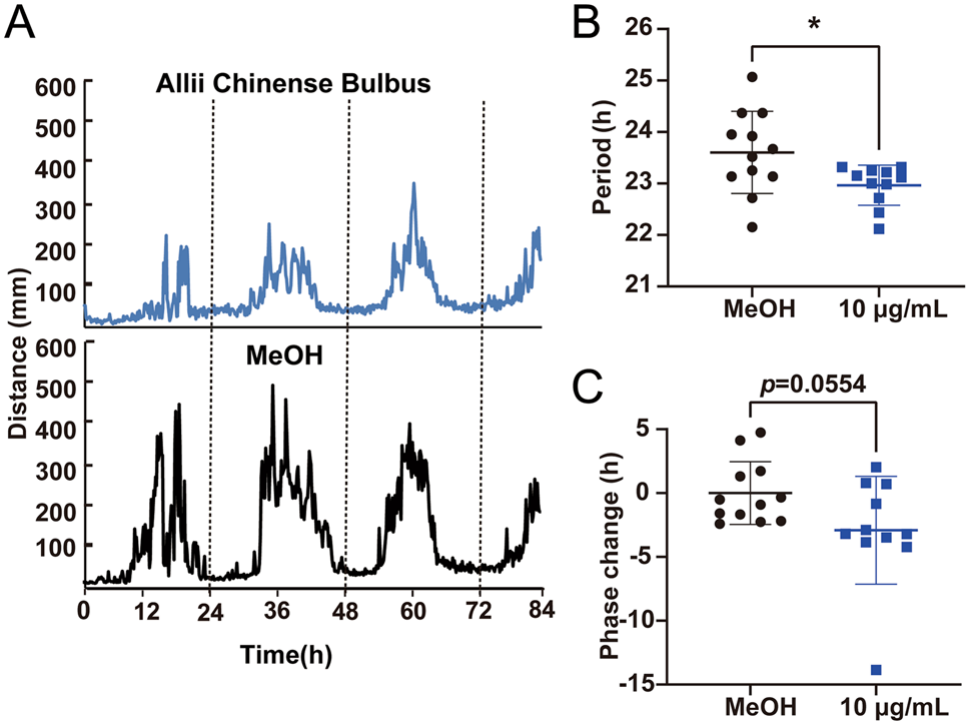
Effects of Allii Chinense Bulbus on locomotor activity rhythms of zebrafish larvae. (**A**) Locomotor activity rhythms under Allii Chinense Bulbus extract (10 µg/mL) and MeOH control. The moving distance (mm) per 10 min were averaged and plotted (n = 11, 12). (**B**,**C**) Effects on circadian period (**B**) and phase (**C**) are shown. Values are average ± SD and were analyzed by Student's t-test (n = 11, 12, *p < 0.05).

To identify circadian clock modulators, 137 crude drug extracts were screened in *Bmal1-dLuc* U2OS for primary screening^7^. Since crude drug extract is a mixture of many active ingredients and their effects could be highly variable, all crude drug extracts were tested in triplicates at two different concentrations (20 µg/mL and 0.20 mg/mL) in a 384-well plate format, and the experiments were repeated twice (Trials 1 and 2) to reduce the number of false positives. Two crude drug (Polygalae Radix and Allii Chinense Bulbus) extracts at 20 µg/mL and four crude drug (Pueraiae Radix, Platycodi Radix, Chrysanthemi Flos, and Paeoniae Radix) extracts at 0.20 mg/mL were identified to show period lengthening effects by one or more hours, respectively (Fig. 1, Table 1). Five crude drug (Artemisiae Capillaris Flos, Polygalae Radix, Perillae Herba, Caryophylli Flos, and Alpiniae Officinari Rhizoma) extracts at 20 µg/mL and eight crude drug (Chrysanthemi Flos, Schizonepetae Spica, Cicadae Periosrtacum, Uncariae Uncis Cum Ramulus, Lonicerae Folium Cum Caulis, Perillae Fruitus, Acorus Graminei Rhizoma, and Mume Fructus Praeparatus) extracts at 0.20 mg/mL were identified to show phase changing effects by two or more hours, respectively (Fig. 1, Table 1). To validate the 17 potential hit crude drug extracts, we performed secondary screening for dose-dependency and confirmed the period and/or phase-changing effects for all 17 crude drug extracts (Fig. 2). To determine whether the effects of crude drugs on the circadian clock were reporter gene-, cell type-, or tissue-specific, the effects of three representative hit crude drugs (Artemisaie Capillaris Flos, Perillae Herba, and Allii Chinense Bulbus) were examined in Rat-1 *Per2*-*luc* fibroblasts^9^ (Fig. 3A). The period lengthening and phase changing effects of the extracts of Artemisiae Capillaris Flos and Perillae Herba were consistent with the results obtained for U2OS cells. Interestingly, however, Allii Chinense Bulbus extract showed period-shortening effects. When we examined the effects of these three crude drug extracts in lung explant cultures from *Per2::Luciferase* knockin mice^7^, all three crude drug extracts showed period-lengthening effects (Fig. 3B). We also examined the effect of Allii Chinense Bulbus extract on the locomotor activity rhythm of zebrafish larvae in vivo under constant darkness and observed a period shortening effect and a tendency to phase change (Fig. 4). As we are aware that some of the compounds affect the bioluminescence amplitude through the activation or inhibition of luciferase activity but not through the circadian clock, we did not focus on the circadian amplitude in the present study.

**Table 1:** List of hit crude drugs.

Number	Name of crude drugs	Origin	Lot number	Distributor
3	Artemisiae Capillaris Flos	The dried capitulum of *Artemisia capillaris* Thunberg	0H03M	Daiko
11	Polygalae Radix	The dried root bark of *Polygala tenuifolia* Willdenow	3I17M	Daiko
14	Puerariae Radix	The dried root of *Pueraria montana* var. *lobata* (Willd.) Sanjappa & Pradeep	C36361	Tsumura
19	Platycodi Radix	The dried root of *Platycodon grandiflorum* A. De Candolle	I420115	Uchida
20	Chrysanthemi Flos	The dried capitulum of *Chrysanmum morifolium* Ramatulle	2H28	Daiko
25	Schizonepetae Spica	The dried spike of *Nepeta tenuifolia* Bentham	7J03	Daiko
46	Paeoniae Radix	The dried root of *Paeonia lactiflora* Pallas	5D08M	Daiko
57	Cicadae Periosrtacum	The dried larval exuvia of *Cryptotympana atrata* Stal	5C30	Daiko
60	Perillae Herba	The dried leaves and branches *of Perilla frutescens* Britton var. *crispa* Decaisne	8F16	Daiko
67	Caryophylli Flos	The dried flowering bud of *Syzygium aromaticum* Merrill et Perry	2G31M	Daiko
68	Uncariae Uncis Cum Ramulus	The dried hook-bearing stem of *Uncaria rhynchophylla* Miquel ex Haviland	1010C022701	Tochimoto
79	Lonicerae Folium Cum Caulis	The dried leaves and stems of *Lonicera japonica* Thunberg	5I21	Daiko
105	Alpiniae Officinari Rhizoma	The dried rhizome of *Alpinia officinarum* Hance	023008001	Tochimoto
118	Perillae Fructus	The dried fruit of *Perilla frutescens* Britton var. *crispa* Decaisne	8A09	Daiko
119	Acorus Graminei Rhizoma	The dried rhizome of *Acorus gramineus* Aiton	P010701311	Tochimoto
129	Mume Fructus Praeparatus	The dried steamed fruit of *Prunus mume* Siebold et Zuccarini	9D01	Daiko
137	Allii Chinense Bulbus	The bulbs of *Allium chinense* G.Don	7I18	Daiko

All crude drugs are registered in the Japanese Pharmacopoeia 17th Edition or the Japanese standards for non-Pharmacopoeial crude drugs, 2015. Daiko Shoyaku (Nagoya, Japan), Tsumura (Tokyo, Japan), Uchida Wakanyaku (Tokyo, Japan), and Tochimoto Tenkaido (Osaka, Japan).

### Ingredients of hit crude drugs modulate the circadian rhythm

**Figure 5:**
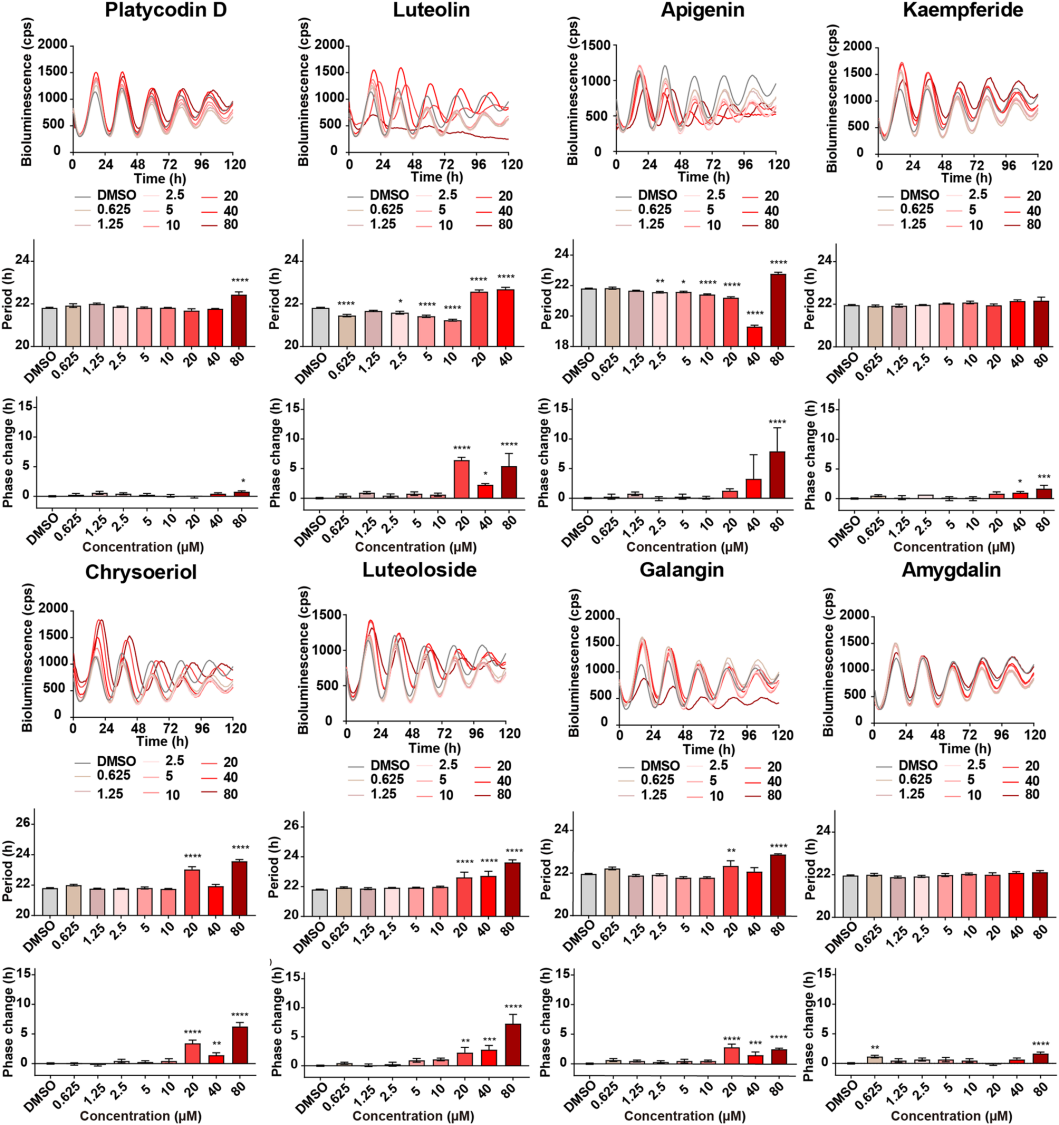
Effects of 8 major ingredients of hit crude drugs. Luminescent traces (top) and dose-dependent effects on the circadian period (middle) and phase (bottom) in U2OS cells. Values are the mean of six replicates ± SD and were analyzed using one way ANOVA, followed by Dunnett’s multiple comparisons test (*p < 0.05, **p < 0.01, ***p < 0.001, **** *p* < 0.0001).

As previously mentioned, the crude drug extract is a mixture containing many active ingredients. In addition, because the administration of crude lysates sometimes affects the experimental results owing to the formation of macromolecular complexes and/or medium pH change^10^, validation using known active ingredients is important. The well-known active ingredients for hit crude drugs are summarized in Table S1. To further understand the possible mode of action of these crude drugs, we searched for known targets of these active ingredients from previous literature and found that AKT, NFκB, and mTOR are the most common targets for these active ingredients (Table S1). To test whether the major ingredients that target AKT, NF-κB, and mTOR can modulate the circadian rhythm, the effects of eight major ingredients were examined in U2OS cells, and it was observed that treatment with these ingredients leads to significant changes in the circadian period and/or phase (Fig. 5).

### Involvement of AKT signaling in the circadian clock

**Figure 6:**
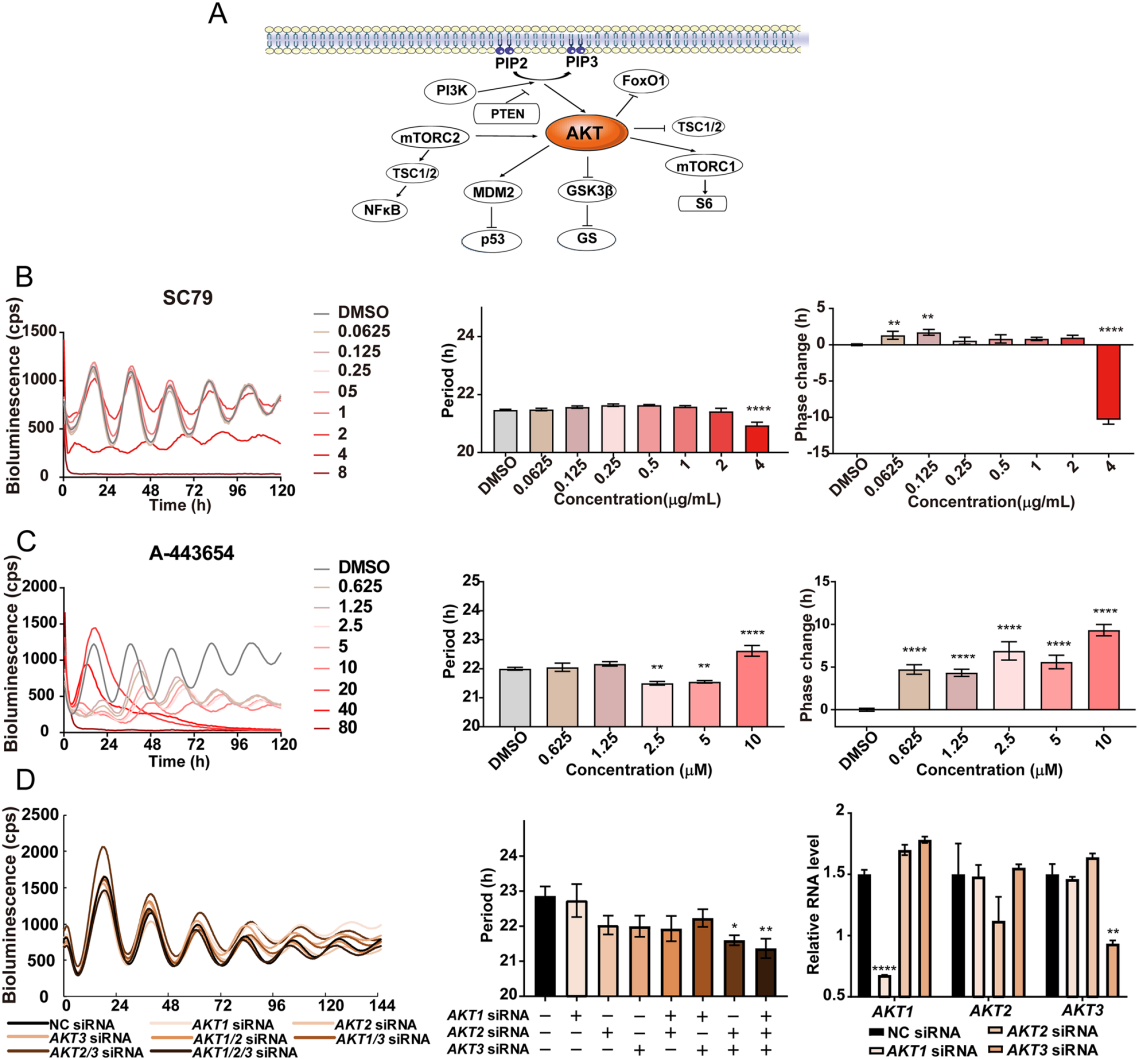
Involvement of AKT signaling in the circadian rhythm. (**A**) Schematic of interactions of the AKT pathway. (**B**,**C**) Effects of AKT activator SC79 (**B**) and AKT inhibitor A-443654 (**C**) at different concentration. Luminescent traces (left) and dose-dependent effects on period (middle) and phase (right) in U2OS cells. (**D**) Effects of the knockdown of *AKT1/2/3* by siRNA. U2OS cells were transfected with negative control (NC) siRNA, *AKT1* siRNA, *AKT2* siRNA, or *AKT3* siRNA. Luminescent traces (left) and effects on circadian period (middle) in U2OS cells. Efficiency of knockdown of *AKT* was confirmed using RT-qPCR (right). Values are the averages of six replicates ± SD and were analyzed using one way ANOVA, followed by Dunnett’s multiple comparisons test (*p < 0.05, **p < 0.01, ***p < 0.001, **** *p* < 0.0001).

It is well established that the signaling pathways for NF-κB, mTOR, and AKT are closely related, and AKT is a major signaling hub (Fig. 6A). Involvement of *Akt* in the circadian clock has been reported in *Drosophila*^11^. There are three isoforms of AKT in mammals. However, analysis of the circadian behavior in *Akt*1-/- mice revealed a normal behavioral rhythm^12^. We examined the effects of AKT activator and inhibitor to explore the role of AKTs in the mammalian circadian clock. The AKT activator SC79 shortened the circadian period and advanced the phase at the highest dose (Fig. 6B). In contrast, the AKT inhibitor A-443654 shortened the circadian period at lower doses and lengthened the period at the highest dose (Fig. 6C). A-443654 also delayed the circadian phase in a dose-dependent manner (Fig. 6C). Furthermore, an siRNA-mediated triple knockdown of *AKT1/2/3* shortened the circadian period (Fig. 6D).

## Discussion

Chronic disruption of the circadian clock due to shift work or travel across time zones has long-term consequences on human health, resulting in an increased risk of many diseases such as diabetes, cardiovascular disease, and cancer^3–6^. The development of new pharmaceuticals is costly and time-consuming, with a high rate of failure. Identifying new functions for existing drugs, known as the drug repositioning approach, is a popular and powerful approach, and crude drugs used in traditional Japanese Kampo medicine are major sources of new chemical entities for drug discovery^7,8^. Indeed, Motohashi et al. succeeded in identifying circadian clock modulators after screening 40 crude drugs^13^.

In this study, we screened 137 crude drug extracts, of which approximately 12% modulated circadian rhythms (Figs. 1 and 2, Table 1). In our previous study, using the US Food and Drug Administration (FDA)-approved drug library and the International Drug Collection (IDC) library, we found that approximately 5% of the existing drugs alter circadian rhythms^7^. The hit rate of Kampo medicines was much higher than that of other existing drug libraries. The reason for the high hit rate is probably because the crude drug extract is a mixture containing many active ingredients. Indeed, the effects of the major ingredients were not as strong as those of the original crude drugs (Fig. 5).

To further test whether these effects are specific to the reporter gene (*Bmal1-dLuc*) or cell type (U2OS cells), we examined the effects of three representative hit crude drugs (Artemisiae Capillaris Flos, Perillae Herba, and Allii Chinense Bulbus) in Rat-1 fibroblasts expressing *Per2-luc* and lung explants from *Per2::Luciferase* knockin mice (Fig. 3). Although the effects of the extracts of Artemisiae Capillaris Flos and Perillae Herba were consistent with those observed in U2OS cells, Allii Chinense Bulbus extract showed the opposite period-shortening effects in Rat-1 cells (Fig. 3). When we examined the effects of Allii Chinense Bulbus extract on the locomotor rhythm of zebrafish larvae in vivo, we observed period shortening effects, as in the case of Rat-1 cells (Fig. 4). Discrepancies in the effects of crude drugs (i.e., period shortening or lengthening) between different reporters and cell types could be a result of the cell type-specific function of the circadian clock gene^14^ and/or the dissociation of clock gene rhythmicity within the cells/tissues^15^. However, as crude drug extracts are a mixture containing many active ingredients, it is also possible that the expression level of the drug targets could be different between different cells and tissues. Notably, tissue-dependent effects of crude drug extracts have also been observed^13^. This study clearly demonstrated the circadian clock modulating effects of crude drugs at the cellular, tissue, and organism levels.

When we searched for the potential targets of the identified ingredients in previous literature, we noticed that most of the ingredients targeted the NF-κB, mTOR, or AKT signaling pathways (Table S1). Among them, AKT is known to be a major signaling hub (Fig. 6A). It is noteworthy that Akt signaling has been shown to affect the circadian period in *Drosophila*^11^. However, there are three isoforms of AKT in mammals, and *Akt1*-/- mice show normal behavioral rhythms^12^. In the present study, we found period and phase changes induced by AKT activator and inhibitor. We also confirmed a significant change in the circadian period in the triple knockdown of *AKT* isoforms, suggesting the involvement of AKT signaling in the mammalian circadian clock (Fig. 6). Notably, PI3K, one of the signaling molecules upstream of the AKT signaling pathway, regulates feeding-mediated entrainment of the peripheral clock^16^. Furthermore, the CLOCK protein has recently been reported to be the substrate of AKT^17^. Further analysis of the precise mode of action of Kampo medicines may uncover the role of the AKT signaling pathway in the circadian clock.

In conclusion, the drug repositioning approach is a useful approach for understanding the underlying mechanisms of the circadian clock. Furthermore, tenuifolin, one of the main active ingredients in Polygalae Radix, has been reported to enhance sleep in mice^18^. Thus, a drug repositioning approach using Kampo medicines is a useful approach for identifying potential therapeutic treatments for circadian misalignment.

## Materials and methods

### Crude drugs

We selected 137 crude drugs that are frequently used as ingredients in traditional Japanese Kampo formulations, and purchased crude drugs that met the grade standards of the Japanese Pharmacopoeia 17th Edition or of non-pharmacopoeial crude drugs from Daiko Shoyaku (Nagoya, Japan), Tsumura (Tokyo, Japan), Uchida Wakanyaku (Tokyo, Japan), and Tochimoto Tenkaido (Osaka, Japan). The preparation of methanol extracts was described in our previous report^8^. Each extract was suspended in methanol at a concentration of 100 mg/mL, and stored at − 20 °C. The origins and the distributors of the hit crude drugs are listed in Table 1.

### Chemicals

Platycodin D (cat no. CFN98134), luteolin (cat no. CFN98784), luteoloside (cat no. CFN98565), galangin (cat no. CFN98918), kaempferide (cat no. CFN98782), and chrysoeriol (cat no. CFN98785) were purchased from ChemFaces (Wuhan, China). Amygdalin (cat no. A0443-1G) was purchased from Tokyo Chemical Industry (Tokyo, Japan). Apigenin (cat no. 010-18914) was obtained from Fujifilm Wako Pure Chemicals (Osaka, Japan). The AKT inhibitor, A-443654 (cat no. HY-10425), and the activator, SC79 (cat no. HY-18749) were purchased from MedChemExpress (Monmouth Junction, NJ, USA). Each chemical was suspended in DMSO at a concentration of 5 mM, stored at − 20 °C.

### Cell culture

Human U2OS cell (HTB-96) and Rat-1 cell were obtained from ATCC (American Type Culture Collection). Human U2OS cells containing a *Bmal1‐dLuc* reporter gene and Rat-1 cells containing *Per2-Luc* reporter gene were cultured in Dulbecco's minimum essential medium (DMEM; D2902, Sigma-Aldrich, St. Louis, MI, USA) supplemented with 10% fetal calf serum (FBS,

FB-1290/500; Biosera, Rue Lacaille, Nuaillen, France), 2 mM L‐glutamine, 100 U/mL penicillin, and 100 μg/mL streptomycin (Pen Strep, 15070063, Thermo Fisher Scientific, Waltham, MA, USA) as previously described^7^.

### Chemical screening

U2OS cells were seeded at a density of 4,000 cells/well in 384‐well plates and incubated for 2 days until confluent. Using a Caliper Life Sciences ALH 3000 Workstation, crude drug extracts were diluted to 20 µg/mL and 0.20 mg/mL respectively, in assay media for measuring luminescence. This resulted in a final MeOH concentration of 0.2% in the control and drug‐treated samples. Multiple doses (0.63, 1.3, 2.5, 5.0, 10, 20, 40, and 80 μM) of ingredients and AKT inhibitor A-443654 were analyzed, while AKT activator SC79 was analyzed at eight different doses (0.063, 0.13, 0.25, 0.5, 1.0, 2.0, 4.0, 8.0 μg/mL). Luminescent assay media (also called “air” or recording media) was composed of DMEM, 10 mM HEPES, 3.5 g/L D‐glucose, 0.35 g/L sodium bicarbonate, 100 U/mL penicillin, 100 μg/mL streptomycin, 2% B27 (Life Technologies, Carlsbad, CA, USA), 0.1 mM luciferin (Fujifilm), and 100 nM dexamethasone (Sigma). Diluted extracts were pipetted into triplicate wells of a 384‐well plate containing U2OS cells (prepared above), and bioluminescence was monitored for 1 week using a Churitsu CL384 Series luminometer (Churitsu Electric Corporation, Nagoya, Japan). The circadian period and phase were determined using NINJA SL00‐01 software for time series analysis (Churitsu Electric Corporation), and potential hit compounds were identified based on changes in the circadian period and/or phase.

### Lung explant culture

Lungs were dissected from male *Per2::Luciferase* knockin mice and cultured in 35 mm dishes containing Millicell cell culture inserts (Millipore) in assay media to measure luminescence, as previously described^7^. The dishes were sealed with silicon grease and parafilm and bioluminescence was measured using a LumiCycle 32 (Actimetrics). The circadian period and phase were determined using LumiCycle Analysis software (Actimetrics). Note that phase-delays and -advances are indicated by positive and negative values, respectively.

### Zebrafish behavior assay

Zebrafish (*Danio rerio*) larvae (3.5 dpf) were placed in each well of a 96 deep-well plate (96 Well Clear Assay Plate Pyramid Flat Bottom); 800 μL E3 medium (5 mM NaCl, 0.17 mM KCl, 0.33 mM CaCl_2_· 2H_2_O, 0.33 mM MgSO_4_· 7H_2_O, 10 mM HEPES, pH 7.2) containing the compound (10 μg/mL) was added to each well. The plate was set on DanioVision (Noldus) and the behavior of the fish was tracked under constant darkness with a CCD camera with infrared light. The amount of activity (i.e., distance moved) per minute was calculated using the image processing function of EthoVision XT11 (Noldus). The free-running period and phase were calculated using Actogram J and MultiCycle (Actimetrics), respectively^19^. All animal studies were carried out in accordance with ARRIVE guidelines and all methods were in compliant with relevant guidelines and regulations and were approved by the Animal Experiment Committee of Nagoya University.

### siRNA mediated knockdown

For gene knockdown analysis, small interfering RNA (siRNA) targeting the *AKT1*, *AKT2*, and *AKT3* sequences and non-targeting siRNA were obtained from Qiagen (FlexiTube GeneSolution siRNA, Hilden, Germany). These siRNAs were introduced singly, or as a mixture of two or three siRNAs into U2OS cells using a reverse transfection protocol in 35 mm dishes. Bioluminescence was monitored in a LumiCycle 32 (Actimetrix, Wilmette, IL, USA) for 1 week. The knockdown effect was verified using quantitative PCR. Reverse transcription was performed on total RNA (200 ng) using ReverTra Ace (Toyobo, Osaka, Japan) and oligo-dT primers. Samples contained SYBR Premix Ex Taq II (Takara, Kusatsu, Japan), 0.4 μM gene-specific primers (Table S2) and 2 µL synthesized cDNA in 20 µL. qPCR was performed on an Applied Biosystems QuantStudio 3 Real-Time PCR System (Foster City, CA, USA) as follows: 95 °C for 30 s, followed by 40 cycles of 95 °C for 5 s and 60 °C for 30 s. ΔCt was determined using *GAPDH* as a housekeeping gene, and relative expression was calculated using the ΔΔCt method by comparing gene‐specific siRNA samples to the negative siRNA control.

### Statistical analysis

Statistical analysis was conducted using GraphPad Prism 9. Significance of differences was analyzed using one way analysis of variance (ANOVA) with Dunnett’s multiple comparisons test or Student's t-test. Values are expressed as mean ± SD.

## Supplementary Information

Supplementary Information 1.

Supplementary Information 2.
