# Supplementary material for: Modulation of circadian clock by crude drug extracts used in Japanese Kampo medicine
Source: Sci Rep. 2021 Oct 26;11:21038. doi: 10.1038/s41598-021-00499-w (PMC8548592; doi:10.1038/s41598-021-00499-w)
Supplement: Supplementary file 2 — Supplementary Information 2. [file 41598_2021_499_MOESM2_ESM.pdf]

Modulation of circadian clock by crude drug extracts used in Japanese Kampo medicine

Manhui Zhang, Kohei Kobayashi, Haruki Atsumi, Yuma Katada, Yusuke Nakane, Junfeng Chen, Ryo Nagano, Naoya Kadofusa, Taeko Nishiwaki-Ohkawa, Naohiro Kon, Tsuyoshi Hirota, Ayato Sato, Toshiaki Makino, and Takashi Yoshimura

References for Supplementary Table 1

1. Masuda, Y., Asada, K., Satoh, R., Takada, K. & Kitajima, J. Capillin, a major constituent of Artemisia capillaris Thunb. flower essential oil, induces apoptosis through the mitochondrial pathway in human leukemia HL-60 cells. *Phytomedicine* **22,** 545552 (2015).
2. Kim, M., Chun, J., Jung, H. A, Choi, J. S. & Kim Y. S. Capillarisin attenuates exercise-induced muscle damage through MAPK and NF-κB signaling. *Phytomedicine* **32**, 30-36 (2017).
3. Kim, J., Lim, J., Kang, B. Y., Jung, K. & Choi, H. J. Capillarisin augments anti-oxidative and anti-inflammatory responses by activating Nrf2/HO-1 signaling. *Neurochem. Int.* **105,** 11-20 (2017).
4. Liu, B. *et al.* Scoparone alleviates inflammation, apoptosis and fibrosis of non-alcoholic steatohepatitis by suppressing the TLR4/NF-κB signaling pathway in mice. *Int. Immunopharmacol.* **75,** 105797 (2019).
5. Chen, S. & Jia, J. Tenuifolin attenuates amyloid-β42-induced neuroinflammation in microglia through the NF-κB signaling pathway. *J. Alzheimers Dis.* **76**, 195-205 (2020).
6. Chen, C. Y., Wei, X. D. & Chen, C. R. 3,4,5-Trimethoxycinnamic acid, one of the constituents of polygalae radix exerts anti-seizure effects by modulating GABAAergic systems in mice. *J. Pharmacol. Sci.* **131**, 1-5 (2015).
7. Askari, V. R. β-Amyrin, the cannabinoid receptors agonist, abrogates mice brain microglial cells inflammation induced by lipopolysaccharide/interferon-γ and regulates Mφ1/Mφ2 balances. *Biomed. Pharmacother.* **101,** 438-446 (2018).

8. Zhang, D. & Li, M. Puerarin prevents cataract development and progression in diabetic rats through Nrf2/HO-1 signaling. *Mol. Med. Rep.* **20,** 1017-1024(2019).
9. She, S., Liu, W., Li, T. & Hong, Y. Effects of puerarin in STZ-induced diabetic rats by oxidative stress and the TGF-β1/Smad2 pathway. *Food Funct.* **5**, 944-950 (2014).
10. Wang, J. F. *et al.* Puerarin protects rat brain against ischemia/reperfusion injury by suppressing autophagy via the AMPK-mTOR-ULK1 signaling pathway. *Neural Regen. Res.* **13**, 989-998 (2018).
11. Rooke, N., Li, D. J., Li, J. & Keung, W. M. The mitochondrial monoamine oxidase-aldehyde dehydrogenase pathway: A potential site of action of daidzin. *J. Med. Chem.* **43,** 4169-4179 (2000).
12. Wei, G., Liang, T., Wei, C., Nong, X., Lu, Q. & Zhao, J. Daidzin inhibits RANKL-induced osteoclastogenesis in vitro and prevents LPS-induced bone loss in vivo. *J. Cell Biochem.* **120**, 5304-5314 (2018).
13. Yang, M. H. *et al.* Attenuation of STAT3 signaling cascade by daidzin can enhance the apoptotic potential of bortezomib against multiple myeloma. *Biomolecules* **10**, 23 (2019).
14. Gu, M. *et al*. Cardioprotective effects of genistin in rat myocardial ischemia-reperfusion injury studies by regulation of P2X7/NF-κB pathway. *Evid.Based Complement. Alternat. Med.* **2016,** 1-9 (2016).
15. Liu, D. *et al*. Genistein acutely stimulates insulin secretion in pancreatic beta-cells through a cAMP-dependent protein kinase pathway. *Diabetes* **55**, 1043-1050 (2006).
16. Choi, J. H. *et al*. Platyconic acid A, platycodi radix-derived saponin, suppresses TGF-β1-Induced activation of hepatic stellate cells via blocking SMAD and activating the PPARγ signaling pathway. *Cells* **8,** 1544 (2019).
17. Li, T. *et al*. Novel Hsp90 inhibitor platycodin D disrupts Hsp90/Cdc37 complex and enhances the anticancer effect of mTOR inhibitor. *Toxicol. Appl. Pharmacol.* **330,** 65-73 (2017).
18. Li, T. *et al*. Platycodin D potentiates proliferation inhibition and apoptosis induction upon AKT inhibition via feedback blockade in non-small cell lung cancer cells. *Sci. Rep.* **6,** 37997 (2016).
19. Kim, H. L., Park, J., Jung, Y., Ahn, K. S. & Um. J. Y. Platycodin D, a novel activator of AMP-activated protein kinase, attenuates obesity in db/db mice via regulation of adipogenesis and thermogenesis. *Phytomedicine* **52**, 254-263 (2019).

20. Zhang, Z., Zhao, M., Zheng, W. & Liu, Y. Platycodin D, a triterpenoid saponin from platycodon grandiflorum, suppresses the growth and invasion of human oral squamous cell carcinoma cells via the NF-κB pathway. *J. Biochem. Mol. Toxicol.* **31,** e21934 (2017).
21. Brusco, I. *et al*. α-Spinasterol: a COX inhibitor and a transient receptor potential vanilloid 1 antagonist presents an antinociceptive effect in clinically relevant models of pain in mice. *Br. J. Pharmacol.* **174**, 4247-4262 (2017).
22. Meng, G., Chai, K., Li, X., Zhu, Y. & Huang, W. Luteolin exerts pro-apoptotic effect and anti-migration effects on A549 lung adenocarcinoma cells through the activation of MEK/ERK signaling pathway. *Chem. Biol. Interact.* **257**, 26-34 (2016).
23. Lee, W. J., Wu, L. F., Chen, W. K., Wang, C. J. & Tseng, T. H. Inhibitory effect of luteolin on hepatocyte growth factor/scatter factor-induced HepG2 cell invasion involving both MAPK/ERKs and PI3K-Akt pathways. *Chem. Biol. Interact.* **160**, 123-133 (2006).
24. Ong, C. S., Zhou, J., Ong, C. N., & Shen, H. M. Luteolin induces G1 arrest in human nasopharyngeal carcinoma cells via the Akt-GSK-3β-Cyclin D1 pathway. *Cancer Lett.* **298**, 167-175 (2010).
25. Shi, R. *et al*. Luteolin sensitizes the anticancer effect of cisplatin via c-Jun NH2-terminal kinase-mediated p53 phosphorylation and stabilization. *Mol. Cancer Ther.* **6**, 1338-1347 (2007).
26. Bao, Y. Y., Zhou, S. H., Lu, Z. J., Fan, J. & Huang, Y. P. Inhibiting GLUT-1 expression and PI3K/Akt signaling using apigenin improves the radiosensitivity of laryngeal carcinoma in vivo. *Oncol. Rep.* **34,** 1805-1814 (2015).
27. Zhang, X. *et al*. The antidepressant effects of apigenin are associated with the promotion of autophagy via the mTOR/AMPK/ULK1 pathway. *Mol. Med. Rep.* **20**, 2867-2874 (2019).
28. Liu, Y. *et al*. Effects of apigenin pretreatment against renal ischemia/reperfusion injury via activation of the JAK2/STAT3 pathway. *Biomed. Pharmacother.* **95**, 1799-1808 (2017).
29. Limboonreung, T., Tuchinda, P. & Chongthammakun, S. Chrysoeriol mediates mitochondrial protection via PI3K/Akt pathway in MPP+ treated SH-SY5Y cells. *Neurosci. Lett.* **714,** 134545 (2020).
30. Wei, W., He, J., Ruan, H. & Wang, Y. In vitro and in vivo cytotoxic effects of chrysoeriol in human lung carcinoma are facilitated through activation of autophagy,

sub-G1/G0 cell cycle arrest, cell migration and invasion inhibition and modulation of MAPK/ERK signaling pathway. *J. BUON.* **24**, 936-942 (2019).

31. Wu, J. Y. *et al*. Chrysoeriol ameliorates TPA-induced acute skin inflammation in mice and inhibits NF-κB and STAT3 pathways. *Phytomedicine* **68,** 153173 (2020).
32. Chen, X. *et al*. Diosmetin induces apoptosis and enhances the chemotherapeutic efficacy of paclitaxel in non-small cell lung cancer cells via Nrf2 inhibition. *Br. J. Pharmacol.* **176,** 2079-2094 (2019).
33. Li, Q. *et al*. Luteoloside attenuates neuroinflammation in focal cerebral ischemia in rats via regulation of the PPARγ/Nrf2/NF-κB signaling pathway. *Int. Immunopharmacol.* **66**, 309-316 (2019).
34. Zhou, M., Shen, S., Zhao, X. & Gong X. Luteoloside induces G0/G1 arrest and pro-death autophagy through the ROS-mediated AKT/mTOR/p70S6K signalling pathway in human non-small cell lung cancer cell lines. *Biochem. Biophys. Res. Commun.* **494**, 263-269 (2017).
35. Shao, J. *et al*. Luteoloside inhibits proliferation and promotes intrinsic and extrinsic pathway-mediated apoptosis involving MAPK and mTOR signaling pathways in human cervical cancer cells. *Int. J. Mol. Sci.* **19,** 1664 (2018).
36. Li, Y. C. *et al*. Paeoniflorin ameliorates fructose-induced insulin resistance and hepatic steatosis by activating LKB1/AMPK and AKT pathways. *Nutrients* **10,** 1024 (2018).
37. Ma, X. H. *et al*. Neuroprotective effect of paeoniflorin on okadaic acid-induced tau hyperphosphorylation via calpain/Akt/GSK-3β pathway in SH-SY5Y cells. *Brain Res.* **1690**, 1-11(2018).
38. Sun, X. *et al*. Paeoniflorin ameliorates cognitive dysfunction via regulating SOCS2/IRS-1 pathway in diabetic rats. *Physiol. Behav.* **174**, 162-169 (2017).
39. Ji, L. *et al*. Paeoniflorin inhibits activation of the IRAK1-NF-κB signaling pathway in peritoneal macrophages from lupus-prone MRL/lpr mice. *Microb. Pathog.* **124**, 223-229 (2018).
40. Yu, J., Xiao, Z., Zhao, R., Lu, C. & Zhang, Y. Paeoniflorin suppressed IL-22 via p38 MAPK pathway and exerts anti-psoriatic effect. *Life Sci.* **180**, 17-22 (2017).
41. Zhang, Y. *et al*. Paeoniflorin attenuates cerebral ischemia-induced injury by regulating Ca2+/CaMKII/CREB signaling pathway. *Molecules* **22**, 359 (2017).
42. Chen, J. *et al*. Paeoniflorin ameliorates AGEs-induced mesangial cell injury through inhibiting RAGE/mTOR/autophagy pathway. *Biomed. Pharmacother.* **89**, 1362-1369 (2017).

43. Hu, B. *et al*. Paeoniflorin attenuates inflammatory pain by inhibiting microglial activation and Akt-NF-κB signaling in the central nervous system. *Cell Physiol. Biochem.* **47**, 842-850 (2018).
44. Sun, J., Li, X., Jiao, K., Zhai, Z. & Sun, D. Albiflorin inhibits the formation of THP-1-derived foam cells through the LOX-1/NF-κB pathway. *Minerva Med.* **110**, 107-114 (2018).
45. Jeong, M. Y. *et al*. Albiflorin ameliorates obesity by inducing thermogenic genes via AMPK and PI3K/AKT in vivo and in vitro. *Metabolism* **73,** 85-99 (2017).
46. Jin, Z. L. *et al*. Receptor and transporter binding and activity profiles of albiflorin extracted from radix paeoniae Alba. *Sci. Rep.* **6**, 33793 (2016).
47. Tsukamoto, Y. *et al*. Rosmarinic acid is a novel inhibitor for hepatitis B virus replication targeting viral epsilon RNA-polymerase interaction. *PLoS One* **13**, e0197664 (2018).
48. Chen, H. *et al*. α-Humulene inhibits hepatocellular carcinoma cell proliferation and induces apoptosis through the inhibition of Akt signaling. *Food Chem. Toxicol*. **134**, 110830 (2019).
49. Rogerio, A. P., Andrade, E. L., Leite, D. F., Figueiredo, C. P. & Calixto, J. B. Preventive and therapeutic anti-inflammatory properties of the sesquiterpene alpha-humulene in experimental airways allergic inflammation. *Br. J. Pharmacol.* **158**, 1074-1087 (2009).
50. Geng, C. A. et al. Antidepressant potential of uncaria rhynchophylla and its active flavanol, catechin, targeting melatonin receptors. *J. Ethnopharmacol.* **232**, 39-46 (2018).
51. Long, H., Ruan, J., Zhang, M., Wang, C. & Huang, Y. Rhynchophylline attenuates tourette syndrome via BDNF/NF-κB pathway in vivo and in vitro. *Neurotox. Res.* **36**, 756-763 (2019).
52. Hu, S. *et al*. Neuroprotection against MPP+-induced cytotoxicity through the activation of PI3-K/Akt/GSK3β/MEF2D signaling pathway by rhynchophylline, the major tetracyclic oxindole alkaloid isolated from uncaria rhynchophylla. *Front. Pharmacol.* **9**, 768 (2018).
53. Lai, T. *et al*. Rhynchophylline attenuates migraine in trigeminal nucleus caudalis in nitroglycerin-induced rat model by inhibiting MAPK/NF-κB signaling. *Mol. Cell. Biochem.* **461**, 205-212 (2019).

54. Zhang, Y. *et al*. Isorhynchophylline enhances Nrf2 and inhibits MAPK pathway in cardiac hypertrophy. *Naunyn Schmiedebergs Arch Pharmacol.* **393**, 203-212 (2019).
55. Xian, Y. F. *et al*. Isorhynchophylline exerts antidepressant-like effects in mice via modulating neuroinflammation and neurotrophins: Involvement of the PI3K/Akt/GSK-3β signaling pathway. *FASEB J.* **33**, 10393-10408 (2019).
56. Liu D. *et al*. Flos lonicerae flavonoids attenuate experimental ulcerative colitis in rats via suppression of NF-κB signaling pathway. *Naunyn Schmiedebergs Arch. Pharmacol.* **393,** 2481-2494 (2020).
57. Gao, L. *et al*. Isorhamnetin protects against cardiac hypertrophy through blocking PI3K-AKT pathway. *Mol. Cell. Biochem.* **429**, 167-177 (2017).
58. Lu, X. *et al*. Isorhamnetin: A hepatoprotective flavonoid inhibits apoptosis and autophagy via P38/PPAR-α pathway in mice. *Biomed. Pharmacother.* **103**, 800-811 (2018).
59. Yang, J. H. *et al*. Isorhamnetin attenuates liver fibrosis by inhibiting TGF-β/Smad signaling and relieving oxidative stress. *Eur. J. Pharmacol*. **783**, 92-102 (2016).
60. Liu, N. *et al*. Isorhamnetin inhibits liver fibrosis by reducing autophagy and inhibiting extracellular matrix formation via the TGF-β1/Smad3 and TGF-β1/p38 MAPK pathways. *Mediators Inflamm*. **2019**, 6175091 (2019).
61. Kim, S. Y. *et al*. Isorhamnetin alleviates lipopolysaccharide-induced inflammatory responses in BV2 microglia by inactivating NF-κB, blocking the TLR4 pathway and reducing ROS generation. *Int. J. Mol. Med.* **43**, 682-692 (2019).
62. Kim, J. E. *et al*. Isorhamnetin suppresses skin cancer through direct inhibition of MEK1 and PI3-K. *Cancer Prev. Res.* **4,** 582-591 (2011).
63. Choi, M. J. *et al*. Anti-inflammatory mechanism of galangin in lipopolysaccharide-stimulated microglia: Critical role of PPAR-γ signaling pathway. *Biomed. Pharmacother.* **144**, 120-131 (2017).
64. Yang, C. C., Lin, C. C., Hsiao, L.D. & Yang, C.M. Galangin inhibits thrombin-induced MMP-9 expression in SK-N-SH cells via protein kinase-dependent NF-κB phosphorylation. *Int. J. Mol. Sci.* **19**, 4084 (2018).
65. Wang, H. B. *et al*. Galangin ameliorates cardiac remodeling via the MEK1/2-ERK1/2 and PI3K-AKT pathways. *J. Cell Physiol.* **234**, 15654-15667 (2019).
66. Lu, H., Yao, H., Zou, R., Chen, X. & Xu, H. Galangin suppresses renal inflammation via the inhibition of NF-κB, PI3K/AKT and NLRP3 in uric acid treated NRK-52E tubular epithelial cells. *BioMed Res. Int.* **2019,** 1-10 (2019).

67. Fu, Q., Gao, Y., Zhao, H., Wang, Z. & Wang, J. Galangin protects human rheumatoid arthritis fibroblast-like synoviocytes via suppression of the NF-κB/NLRP3 pathway. *Mol. Med. Rep.* **18**, 3619-3624 (2018).
68. Wang, D. *et al*. Kaemp feride protects against myocardial ischemia/reperfusion injury through activation of the PI3K/Akt/GSK-3β pathway. *Mediators Inflamm.* **2017**, 5278218 (2017).
69. Piermartiri, T., Pan, H., Figueiredo, T. H. & Marini, A. M. α-Linolenic Acid, A nutraceutical with pleiotropic properties that targets endogenous neuroprotective pathways to protect against organophosphate nerve agent-induced neuropathology. *Molecules* **20**, 20355-20380 (2015).
70. Shen, Y., Sun, Z. & Guo, X. Citral inhibits lipopolysaccharide-induced acute lung injury by activating PPAR-γ. *Eur. J. Pharmacol.* **747**, 45-51 (2014).
71. Gonçalves, E. C. D. *et al*. Citral inhibits the inflammatory response and hyperalgesia in mice: The role of TLR4, TLR2/Dectin-1, and CB2 cannabinoid receptor/ATP-sensitive K+ channel pathways. *J Nat Prod.* **83,** 1190-1200 (2020).
72. Wang, T. L., Ouyang, C. S. & Lin, L. Z. β-Asarone suppresses Wnt/β-catenin signaling to reduce viability, inhibit migration/invasion/adhesion and induce mitochondria-related apoptosis in lung cancer cells. *Biomed. Pharmacother.* **106,** 821-830 (2018).
73. Li, L. *et al*. β-Asarone induces apoptosis and cell cycle arrest of human glioma U251 cells via suppression of HnRNP A2/B1-mediated pathway in vitro and in vivo. *Molecules* **23,** 1072 (2018).
74. Lv, L. N. *et al*. β-Asarone increases doxorubicin sensitivity by suppressing NF-κB signaling and abolishes doxorubicin-induced enrichment of stem-like population by destabilizing Bmi1. *Cancer Cell Int.* **19,** 153 (2019).
75. Ning, B., Zhang, Q., Wang, N., Deng, M. & Fang, Y. β-Asarone regulates ER stress and autophagy via inhibition of the PERK/CHOP/Bcl-2/Beclin-1 pathway in 6-OHDA-induced parkinsonian rats. *Neurochem. Res.* **44**, 1159-1166 (2019).
76. Liczbiński, P. & Bukowska, B. Molecular mechanism of amygdalin action in vitro: Review of the latest research. *Immunopharmacol. Immunotoxicol.* **40**, 212-218 (2018).
77. Shi, Y. *et al*. Oleanolic acid induced autophagic cell death in hepatocellular carcinoma cells via PI3K/Akt/mTOR and ROS-dependent pathway. *Korean J. Physiol. Pharmacol.* **20**, 237-243 (2016).

78. Guo, G., Yao, W., Zhang, Q. & Bo, Y. Oleanolic acid suppresses migration and invasion of malignant glioma cells by inactivating MAPK/ERK signaling pathway. *PLoS One* **8**, e72079 (2013).
79. Zhao, H. *et al*. Oleanolic acid rejuvenates testicular function through attenuating germ cell DNA damage and apoptosis via deactivation of NF-κB, p53 and p38 signaling pathways. *J. Pharm. Pharmacol.* **69**, 295-304 (2017).
80. Lu, J. *et al*. Alliin attenuates 1, 3-dichloro-2-propanol-induced lipogenesis in HepG2 cells through activation of the AMP-activated protein kinase-dependent pathway. *Life Sci.* **195**, 19-24 (2018).
81. Wang, Y. L., Guo, X. Y., He, W., Chen, R. J. & Zhuang, R. Effects of alliin on LPS-induced acute lung injury by activating PPARγ. *Microb. Pathog.* **110**, 375-379 (2017).
82. Yang, J. *et al*. Diallyl disulfide alleviates inflammatory osteolysis by suppressing osteoclastogenesis via NF-κB-NFATc1 signal pathway. *FASEB J.* **33**, 7261-7273 (2019).
83. Yue, Z. *et al*. Diallyl disulfide induces apoptosis and autophagy in human osteosarcoma MG-63 cells through the PI3K/Akt/mTOR pathway. *Molecules* **24**, 2665 (2019).
84. Xie, X. *et al*. Diallyl disulfide inhibits breast cancer stem cell progression and glucose metabolism by targeting CD44/PKM2/AMPK signaling. *Current Cancer Drug Targets* **18**, 592-599 (2018).
85. Luo N. *et al*. Induction of apoptosis in human leukemic cell lines by diallyl disulfide via modulation of EGFR/ERK/PKM2 signaling pathways. *Asian Pac. J. Cancer Prev.* **16**, 3509-3515 (2015).
